# Membrane manipulations by the ESCRT machinery

**DOI:** 10.12688/f1000research.6319.1

**Published:** 2015-08-07

**Authors:** Greg Odorizzi

**Affiliations:** 1 Molecular, Cellular, and Developmental Biology, University of Colorado, Boulder, CO, USA

**Keywords:** ESCRT, ESCRT machinery, endosomal sorting complexes required for transport, membrane dynamics, intralumenal vesicle budding pathway, retrovirus budding, cytokinesis

## Abstract

The endosomal sorting complexes required for transport (ESCRTs) collectively comprise a machinery that was first known for its function in the degradation of transmembrane proteins in the endocytic pathway of eukaryotic cells. Since their discovery, however, ESCRTs have been recognized as playing important roles at the plasma membrane, which appears to be the original site of function for the ESCRT machinery. This article reviews some of the major research findings that have shaped our current understanding of how the ESCRT machinery controls membrane dynamics and considers new roles for the ESCRT machinery that might be driven by these mechanisms.

Genes encoding the proteins that came to be known collectively as the endosomal sorting complexes required for transport (ESCRT) machinery were discovered in the yeast *Saccharomyces cerevisiae* ^1^. Genetic screens revealed these genes to be required for the sorting of transmembrane proteins into intralumenal vesicles (ILVs) that bud into the endosome lumen ^2, 3^. Biochemical studies assigned the different yeast gene products into distinct protein complexes that were dubbed ESCRTs ^4– 7^. Although they were defined in yeast, almost every protein subunit that belongs to an ESCRT complex can also be identified in every other eukaryotic genome, although ESCRT-0 appears restricted to metazoans and fungi ^8^, and some orthologs are also found in archaeal species ^9, 10^. Such comprehensive distribution implies that at least some of the functions executed by the ESCRT machinery are highly conserved.

## ESCRTs in the intralumenal vesicle budding pathway

**Figure 1.**
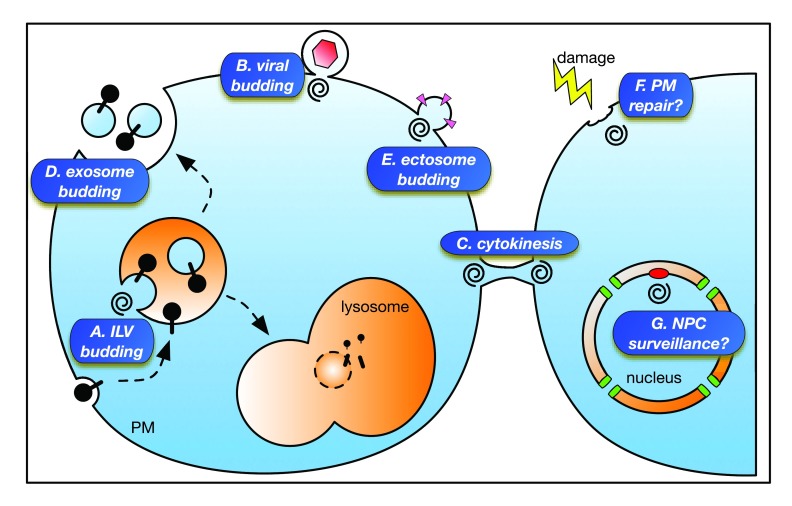
Broad overview of endosomal sorting complex required for transport (ESCRT) activities in membrane dynamics. ESCRTs are generally represented by a spiral, which reflects the conformation adopted by ESCRT-III. ( **A**) In the intralumenal vesicle (ILV) budding pathway, ESCRTs sort transmembrane proteins at endosomes into ILVs that are degraded when endosomes fuse with lysosomes. ( **B**) In the viral budding pathway, ESCRTs are required at the plasma membrane (PM) for the release of infectious viral particles. ( **C**) In the abscission step of cytokinesis, ESCRTs are required at the PM for membrane scission that separates dividing cells. ( **D**) In the exosome budding pathway, ILVs created by ESCRTs at endosomes are released into the extracellular environment when endosomes fuse with the PM. ( **E**) In the ectosome budding pathway, extracellular vesicles are released directly from the PM. Recent studies implicate the ESCRT machinery functioning in the repair of PM damage ( **F**) and in the elimination of dysfunctional nuclear pore complex (NPC) intermediates (denoted in red) from the inner nuclear membrane ( **G**).

A key insight at the time of its discovery was that the ESCRT-I complex binds ubiquitin ^4^. ESCRT-0 was subsequently found to have the same ubiquitin-binding property ^7^, as was ESCRT-II ^11^. Shortly before the ESCRTs were described as ubiquitin-binding protein complexes, studies revealed that many transmembrane proteins at the plasma membrane are ubiquitinated on their cytosolic domains and that this modification was essential for their degradation at lysosomes ^12^. Together, these observations established that one of the functions of the ESCRT machinery is to target ubiquitinated transmembrane proteins for lysosomal degradation by sorting them at endosomes into ILVs, which then are exposed to degradative hydrolytic enzymes when late endosomes fuse with lysosomes ( Figure 1A).

Lagging behind the advances in understanding how ESCRTs recognize ubiquitinated ILV cargoes was insight into how the ILVs are actually created. This impasse was breached 10 years later with the discovery that purified subunits of the yeast ESCRT-III complex, when added to synthetic liposomes, can catalyze the membrane scission reaction required for the detachment of ILVs from the limiting membrane ^13^. This observation explained why dominant-negative alleles that affect ESCRT-III function *in vivo* had been seen in earlier studies to disrupt the budding of retroviruses, a process that is topologically similar to ILV budding and also dependent upon the ESCRT machinery (see below). The same experimental system showed that purified yeast ESCRT-I and ESCRT-II complexes can cooperate with one another to induce the membrane invagination step that initiates ILV budding ^14^. Thus, a broad outline of the division of functions executed by the ESCRT machinery at endosomes was proposed: ESCRT-0, ESCRT-I, and ESCRT-II concentrate ubiquitinated transmembrane proteins at membrane microdomains, where ESCRT-I and ESCRT-II produce incipient buds that are pinched off by the membrane scission activity of ESCRT-III.

Despite the assignment of activities performed by certain ESCRTs that was suggested *in vitro* ^13, 14^, many of the details about their operations remain fuzzy. Perhaps the wooliest thinking concerns ESCRT-III structure-function. What makes it troublesome to study is that ESCRT-III is not a stable protein complex. Instead, its subunits exist in two states that are in equilibrium with one another: subunits either are monomeric and soluble in the cytosol or associate with membranes, where they polymerize into the ESCRT-III complex ^6, 15^. All of the ESCRT-III subunits are homologous to one another and are predicted to have the same three-dimensional (3D) structure, yet structures have been determined for only some of the subunits (either in whole or in part), and these structures were solved either for an individual subunit or for two different subunits bound to one another ^16– 19^ but not for all of the subunits co-assembled together. Macromolecular structures that correspond to ESCRT-III on membranes *in vivo* have been visualized by electron microscopy (EM) ^20, 21^, but no studies have unambiguously solved a structure of the complete ESCRT-III complex in relative isolation. Even the stoichiometry of its subunits has been defined only in relatively loose terms ^22^.

Murkier still is the mechanism by which ESCRT-III drives the membrane scission reaction. This topic has been reviewed often (e.g., 23) and can be boiled down to two working models. One proposes that the polymerization of ESCRT-III subunits mediates scission. This model draws its support from *in vitro* studies, including the original demonstration that purified ESCRT-III subunits assembled on synthetic membranes catalyze membrane scission; in this assay, the disassembly of ESCRT-III was necessary only for the replenishment of subunits so that they can participate in further rounds of complex assembly ^13^. An assembly-driven model for scission is also supported by EM of purified ESCRT-III subunits that, when combined with one another, polymerize into tubules constricted at one end to form a dome shape ^17^. This particular conformation led to the idea that the assembly of membrane-associated subunits at the neck of an ILV invagination would narrow the membrane to terminate in constriction and culminate in scission ^24^, but whether a dome-shaped structure is formed by ESCRT-III *in vivo* is unknown.

The other model contends that membrane scission by ESCRT-III is coupled to disassembly of the complex by VPS4, a member of the broader family of AAA+ ATPases that are known for their roles in the disassembly of macromolecular complexes. This model is supported by studies conducted in intact cells showing that scission is stalled when VPS4 activity is inhibited ^20, 25, 26^. Like other AAA+ ATPases, ATP-bound VPS4 assembles into a ring-shaped oligomer with a central pore. The amino terminus of VPS4 is oriented toward the pore of the oligomer, and this region has a domain that binds directly to a motif located in each ESCRT-III subunit ^9, 27, 28^. Via this interaction, it is thought that VPS4 rips the ESCRT-III complex apart by extruding each individual subunit in succession through its pore when it hydrolyzes ATP. Conceivably, this action could shorten the ESCRT-III complex to gradually draw together the attached membrane in the neck of an ILV constriction, ultimately resulting in scission ^29^. An alternative scenario consistent with studies conducted *in vivo* is that the engagement of VPS4 with ESCRT-III substrates alters the conformation of the ESCRT-III polymer, potentially serving to catalyze the membrane scission reaction ^20, 30, 31^.

Despite uncertainties surrounding the native structure of ESCRT-III *in vivo* and the mechanism by which it drives membrane scission, its function in this process is well supported by studies revealing that the role of ESCRT-III is not restricted to the ILV budding pathway. Indeed, the membrane scission activity of ESCRT-III appears to have essential roles in cellular processes that are unrelated to transmembrane protein degradation. These activities are considered below.

## ESCRT activity in retrovirus budding

Very shortly after they were reported to function in the ILV budding pathway, ESCRT-I and VPS4 were discovered to have a role in the budding of human immunodeficiency virus-1 (HIV-1) from the plasma membrane of infected cells ^32^. In addition to exposing ESCRTs as having non-endosomal functions, this landmark report opened the door for an explosion of studies revealing that a subset of ESCRTs are generally required for the budding of all retroviruses and for many non-retroviral classes of viruses (reviewed in 33). Exploitation of the ESCRT machinery is driven by virally encoded proteins that recruit one or more ESCRT subunits to the membrane microdomain where new viruses are being packaged, the goal being to nucleate a protein interaction network that mediates recruitment of ESCRT-III ^34^. Viral budding critically depends upon ESCRT-III (and VPS4) to catalyze the membrane scission reaction necessary for the release of a virion from the host cell membrane ( Figure 1B). Thus, like the ILV budding pathway, the retroviral budding pathway depends on ESCRT-III/VPS4 at the final step to sever the membrane.

The identities of ESCRTs, aside from ESCRT-III/VPS4, recruited by each retrovirus are idiosyncratic. For instance, HIV-1 recruits both ESCRT-I ^32^ and ALIX, the latter of which interacts with ESCRT-III ^35^, whereas the equine infectious anemia virus recruits only ALIX ^35^. The reason for selective utilization of components outside of the core ESCRT-III/VPS4 machinery might be that viruses do not need all of the activities that are performed by ESCRT complexes 0, I, and II in the ILV budding pathway ^33^. For example, the structural proteins encoded by viruses can target themselves to the site of viral assembly and autonomously generate the membrane curvature needed to produce a virion. That different viruses recruit different ESCRTs to facilitate their exploitation of the membrane scission activity performed by ESCRT-III/VPS4 is a reflection of how evolution allows different solutions for the same problem.

Notably, studies of how viruses take advantage of ESCRTs have yielded fundamental advances in understanding mechanisms that are likely common to all pathways that use ESCRT-III/VPS4. For example, the HIV-1 budding pathway was found to be affected by truncation of ESCRT-III subunits, leading to structure-function analyses demonstrating that the carboxyl termini of several ESCRT-III subunits make intramolecular contacts with their amino-terminal core regions to maintain the proteins as inactive subunits that are incapable of assembling into the ESCRT-III complex ^16, 36, 37^. More recently, 3D super-resolution microscopy and correlative EM outlined the nanoscale organization within the head of budding HIV-1 virions, suggesting that VPS4 functions at least in part to remodel subunits assembled into the ESCRT-III complex during the membrane constriction process that leads to scission ^38^. A direct role for VPS4 in membrane scission during retrovirus budding was also supported by light microscopy studies that tracked HIV-1 recruitment of ESCRTs over time to the plasma membrane of live cells ^39, 40^.

In addition to characterizing the mechanisms of viral exploitation of the ESCRT machinery, investigations of this process helped lead to the unexpected discovery that ESCRT-III/VPS4 has a role during cytokinesis in animal cells, as discussed in the next section.

## ESCRT activities during cytokinesis

In metazoans, dividing daughter cells are connected by a relatively thin (200 nm) membrane tubule known as the midbody. A process termed abscission severs the midbody connection, and this step appears to depend on the membrane scission activity of ESCRT-III/VPS4 ( Figure 1C; reviewed in 41). This discovery originated from serendipitous observations in a number of disparate studies, not all of which were aimed at understanding ESCRT functions per se, and was bolstered by proteomic studies that identified several ESCRT-binding proteins implicated to function at the midbody. Focused analyses of ESCRTs in cytokinesis revealed that the TSG101 subunit of human ESCRT-I binds directly to CEP55, a microtubule bundling protein, and this interaction mediates ESCRT-I recruitment to the midbody ^42– 44^. TSG101 also binds ALIX, the ESCRT-III-associated protein mentioned above ^45, 46^. Colocalization of ALIX with TSG101 to the midbody was found to require CEP55, and, consistent with their function during cytokinesis, knocking down TSG101 or ALIX expression results in multi-nucleated cells, and this is a hallmark cytokinetic defect ^42– 44^.

The point of CEP55 mediating recruitment of TSG101/ALIX appears to be so that ALIX can mediate recruitment of CHMP4B, which is the predominant subunit comprising the ESCRT-III complex. As in the ILV and viral budding pathways, ESCRT-III assembly within the midbody neck is expected to constrict the membrane. Three-dimensional electron tomography of the midbody revealed 17-nm-thick spiral filaments adjacent to membrane constrictions ^21^, analogous to spirals of CHMP4B at the plasma membrane that were observed by deep-etch EM ^20^. However, immunolabeling confirmed that the spirals imaged in the latter study were comprised of CHMP4B, and these spirals measured only 5 nm in thickness ^20^, which is close to the 9-nm-thick spirals of the purified yeast ortholog of CHMP4B that were seen *in vitro* by negative staining EM ^47^ and the 4-nm-thick spirals of the *Caenorhabditis elegans* ortholog of CHMP4B observed *in vitro* by cryo EM ^31^. The severe limitations of immunolabeling in electron tomography made it impossible to confirm that CHMP4B (or other ESCRT-III subunits) comprise the 17-nm spiral filaments in the midbody, although this identity seems likely, given that formation of these filaments was dependent upon expression of a different subunit of the ESCRT-III complex ^21^. Given that several cytoskeletal elements also oligomerize into filaments within the midbody, something else might comprise the 17-nm spiral structures presumed to be ESCRT-III; alternatively, ESCRT-III might co-assemble with something else (or with itself in parallel polymers) to form the 17-nm filaments seen by tomography.

Notwithstanding the uncertainties described above, a role for ESCRT-III/VPS4 in membrane scission during cytokinesis is strongly supported by other lines of evidence. First and foremost is the discovery that orthologs of VPS4 and ESCRT-III subunits mediate cytokinesis during cell division in archaebacterial species ^10, 48^. Thus, the function of ESCRT-III/VPS4 most likely originated for this purpose. Additionally, time-lapse imaging by high-resolution light microscopy showed sequential recruitment of TSG101 and CHMP4B to the intercellular membrane bridge of the midbody connecting daughter cells, followed by VPS4, whereupon cell separation occurs ^49^. Curiously, the kinetics involved during abscission are considerably slower than what has been observed in retroviral budding, potentially to allow for checkpoints that ensure the fidelity of cytokinesis. For instance, the Aurora B kinase that regulates chromosome contacts with microtubules also phosphorylates CHMP4C (a CHMP4B paralog), which causes CHMP4C to concentrate at the midbody ^50^. As a result, abscission is inhibited, possibly signifying that phosphorylated CHMP4C interferes with the function of CHMP4B. Another intriguing possibility is that the timing of scission is regulated by tension: pulling forces between daughter cells were found to prolong their connection, whereas relaxation of this tension coincided with ESCRT-III assembly and subsequent abscission ^51^. A role for tension in functions of the ESCRT machinery that drive the ILV budding pathway has also been modeled by using data derived from a variety of studies ^52^.

## ESCRTs and the biogenesis of extracellular vesicles

Components of the ESCRT machinery have also been linked to the formation of extracellular vesicles (EVs) that are secreted by many (if not all) cell types. EVs serve as shuttles that mediate intercellular exchange of proteins, RNAs, and lipids, and their functions in humans have been shown to have critical physiological roles in the immune, cardiovascular, and nervous systems ^53, 54^. Two types of EVs have been defined, and they can be readily distinguished by the way in which they are secreted. EVs known as exosomes originate as ILVs within endosomes and are released from the cell when late endosomes fuse with the plasma membrane rather than a lysosome ( Figure 1D); EVs known as ectosomes (or shedding vesicles or microvesicles) bud directly from the plasma membrane ( Figure 1E).

A connection between exosome biogenesis and the ILV budding pathway is easy to imagine because they have a common origin, and several proteomic studies had identified ESCRT proteins in purified exosomes (reviewed in 55). However, an ESCRT-independent lipid-driven model for exosome biogenesis had originally been invoked by the observation that ceramide, which resides in the inner leaflet of the exosomal membrane, has a small head group and forms extended hydrogen bond networks that cluster this lipid species into microdomains favorable toward budding into the endosome lumen ^56^. The ESCRT machinery was subsequently realized to have a role in exosome biogenesis when it was discovered that syndecan heparan sulfate proteoglycans (one type of cargo packaged into exosomes) interact via a cytoplasmic adaptor protein with ALIX, and exosome budding is blocked upon knocking down expression of ALIX, VPS4, or CHMP4 subunits of the ESCRT-III complex ^57^. This finding indicates that exosomal cargoes can nucleate exosome budding through recruitment of at least a subset of the ESCRT machinery, analogous to the way in which retroviral proteins recruit ESCRTs to bud from infected cells.

Evidence was also obtained that the ESCRT machinery functions in ectosome biogenesis. One report showed that knocking down VPS4 expression in *C. elegans* inhibits ectosome production ^58^, whereas another showed that a human arrestin domain-containing protein that binds the TSG101 subunit of ESCRT-I is packaged into ectosomes, and the budding of these ectosomes was blocked by knocking down expression of TSG101 or VPS4 ^59^. TSG101 and VPS4 were similarly reported to be required for the release of ectosomes loaded with human T-cell receptors into the immunological synapse ^60^.

Our understanding of the roles played by ESCRTs in the biogenesis of EVs is still in its infancy, and many details need to be worked out. For instance, do all cell types employ the same set of ESCRTs for exosome or ectosome budding (or both)? In ectosome biogenesis, do ESCRTs function in membrane deformation, as they appear to do in the ILV budding pathway ^13^, or do they function predominately in membrane scission? And beyond ESCRTs, what determines whether an endosome fuses with the plasma membrane to release exosomes instead of fusing with a lysosome to deliver ILVs to their destruction?

## A new frontier?

The past year witnessed two discoveries revealing new potential roles for the ESCRT machinery. The repair of small (<100 nm) lesions in the plasma membrane was found to require ESCRT-III ( Figure 1F; 61). In this case, however, it remains unclear whether ESCRT-III has a direct role, as the kinetics of its recruitment to wounded plasma membranes are a few seconds slower than the observed amount of time required for repair to occur ^62, 63^. One possibility is that remodeling of the plasma membrane occurs in response to wounding and that this process depends directly on ESCRT-III function ^64^. More radically, ESCRT-III and VPS4 were found to be required at the inner nuclear membrane for the removal of defective nuclear pore complex (NPC) assembly intermediates ( Figure 1G; 65), but how they might function in this process is unknown. One possibility is that nucleoporins extracted from defective NPCs are packaged by ESCRT-III/VPS4 into vesicles, but nucleoporin degradation mediated by this quality-control pathway appears to involve proteasomal degradation, the substrates of which cannot be membrane-bound. Thus, although it is still too early to tell whether the additional duties ascribed to ESCRTs in plasma membrane wound repair and NPC quality control are mediated by any of the functions already known for the ESCRT machinery, these unexpected findings signal a new frontier of understanding how ESCRTs have evolved from their apparently primordial beginning in cell division.
