# Supplementary material for: Membrane manipulations by the ESCRT machinery
Source: F1000Res. 2015 Aug 7;4(F1000 Faculty Rev):516. [Version 1] doi: 10.12688/f1000research.6319.1 (PMC4544380; doi:10.12688/f1000research.6319.1)
Supplement: Peer review history

# Peer review: Referee response for version 1

**DOI:** 10.5256/f1000research.6776.r9924

**Published:** 2015-08-07

**Authors:** Phyllis Hanson

**Affiliations:** 1 Department of Cell Biology & Physiology, Washington University School of Medicine, St. Louis, MO, USA

I have read this submission. I believe that I have an appropriate level of expertise to confirm that it is of an acceptable scientific standard.

# Peer review: Referee response for version 1

**DOI:** 10.5256/f1000research.6776.r9921

**Published:** 2015-08-07

**Authors:** Jon Audhya

**Affiliations:** 1 Department of Biomolecular Chemistry, University of Wisconsin-Madison, Madison, WI, 53706, USA

I have read this submission. I believe that I have an appropriate level of expertise to confirm that it is of an acceptable scientific standard.

# Peer review: Referee response for version 1

**DOI:** 10.5256/f1000research.6776.r9925

**Published:** 2015-08-07

**Authors:** Winfried Weissenhorn

**Affiliations:** 1 Unit of Virus Host Cell Interactions, University Joseph Fourier, Grenoble, France

I have read this submission. I believe that I have an appropriate level of expertise to confirm that it is of an acceptable scientific standard.

# Peer review: Referee response for version 1

**DOI:** 10.5256/f1000research.6776.r9923

**Published:** 2015-08-07

**Authors:** David Teis

**Affiliations:** 1 Innsbruck Medical University, Innsbruck, Austria

I have read this submission. I believe that I have an appropriate level of expertise to confirm that it is of an acceptable scientific standard.
